# Burnout and Its Antecedents: Considering Both Work and Household Time Claims, and Flexibility in Relation to Burnout

**DOI:** 10.3389/fpubh.2022.863348

**Published:** 2022-05-10

**Authors:** Shuanglong Li, Jannes ten Berge, Marcus H. Kristiansen

**Affiliations:** ^1^Department of Sociology, School of Public Administration, Guangzhou University, Guangzhou, China; ^2^Erasmus School of Social and Behavioural Sciences, Erasmus University Rotterdam, Rotterdam, Netherlands; ^3^Norwegain Tax Authority, Oslo, Norway

**Keywords:** burnout, work and family time claims, flexibility, moderation effect, the Netherlands

## Abstract

This study contributes to the previous literature by examining how flexible work arrangements interact with work and family time claims to affect burnout. It does so by providing a theoretical framework and empirical test of the interaction of flexibility with the effect of work and family time claims on burnout. Hypotheses and predictions based on previous literature are tested by Ordinary Least Squared regression models using data from the Time Competition Survey, constituting a sample of 1,058 employees of 89 function groups within 30 organizations. We found no main effects of work and family time claims or flexible work arrangements on burnout. However, the results do show an interaction of flexible working hours with the effect of work and family time claims on burnout. Specifically, the higher an individual's work and family time claims, the more this person benefits from having flexible working hours. In general, the results support the proposition that the relationship between work and family time claims and burnout differs for individuals with different levels of flexible work arrangements.

## Introduction

The recent decades have witnessed significant changes in labor market conditions and increased precariousness of employment relations. For example, there has been a rise in non-standard employment in developed countries such as part-time work, temporary work, contract work, and gig economy work (1). Previous research has attributed the changed employment relations to a variety of social-economic and cultural factors such as the rise of neoliberalism economic policies, rapid technological advances, and increased ethnic and gender diversity in the labor market (1–3). These non-standard forms of employment are associated with not only low payment, but also with unpredictable work schedules, low collective voice, and employees' poor health and wellbeing (4, 5).

In this context, burnout is a common problem among the employees in today's work organizations (6–9). For example, between 2007 and 2011, the Dutch working force that reported burnout complaints grew from 11 to 13% [CBS (10)]. In the scientific literature, the term burnout was first introduced by the psychotherapist Herbert Freudenberger (11), and ever since the antecedents and consequences of burnout have received much scholarly attention from several social science disciplines [Refer to (12) for an overview]. The concept of burnout may be defined as a state of emotional, physical, and mental exhaustion resulting from occupational stress (13) and is associated with many work-related outcomes, such as extreme exhaustion, detachment from the job, a lack of accomplishment, cynicism, and may also have a negative spillover on co-workers (12, 14). Moreover, burnout is related to mental health problems, such as anxiety, depression, and drops in self-esteem, although the causal direction of this relationship remains unclear (12, 13).

Turning to the antecedents of burnout, one of the main topics of study is how the competing time claims from work (15, 16) and household domains (17, 18) relate to burnout [e.g., (12, 19, 20) for a review]. One of the reasons for this interest is the argued rise in the problem of balancing the time claims from both work and household (21), as a consequence of the increase of single-parent households and dual-earner families (14, 22, 23). That is, families have been moving away from a more “traditional” division of labor between spouses, in which the husband provides the income and the wife takes care of the household and leads individuals to occupy roles both within the household and work sphere. The difficulties that may occur in combining several roles have been related to burnout (24). In particular, this notion is found within the role conflict theory, which asserts that combining and fulfilling several roles causes strain that in turn leads to higher levels of burnout (16, 17, 24–27). On the contrary, the enrichment approach highlights that occupying multiple roles may provide resources for the individual, alleviating burnout (17, 24, 28, 29). Considering the above, it is not readily apparent from the literature whether high work and household time claims lead to lower or higher levels of burnout.

To accommodate employees' fulfillment of both work and family roles, several family-supportive policies, e.g., parental leave, part-time work, and child care facilities, have been implemented (22). In relation to work and family time claims and burnout or related outcomes, two arrangements, in particular, have been studied, such as flexible work schedules (i.e., flextime) and working from home (i.e., flexplace) (30, 31). However, in explaining this relationship, only few studies conceptualize the relation between flexibility and burnout as an interaction with the work and household spheres (30, 32). Moreover, the one study that tests the interaction between time claims and flexibility (30) only has measures for family time claims and employs affective stress as the outcome variable.

In the present study, we aim to contribute to the existing literature by addressing the shortcomings mentioned earlier and examining how flexibility and work and household time claims interact to affect burnout. First, we establish the theoretical link between work and household time claims and burnout. Then flexibility is introduced as a moderator of this relationship, providing a coherent conceptualization of the role of flexibility in explaining burnout. Second, we proceed by addressing the shortage of empirical testing regarding the interaction of flexibility with the effect of work and family time claims on burnout. This is accomplished by including the interaction in our empirical analysis. An additional advantage of the present study and making the former contributions possible is using a unique large-scale dataset from the Netherlands. This dataset, the Time Competition Survey (33), contains detailed information on employees' work and family characteristics from 30 different organizations. Particularly relevant for the study of time claims is the time diary measure of hours spent at work and performing various tasks in the household. The former contributions are summarized in the following research question: How is the effect of work and family time claims on burnout moderated by flexibility?

## Theories and Hypotheses

**Figure 1:**
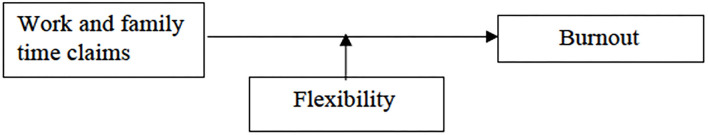
The effect of work and family time claims on burnout and the moderating effect of flexibility.

This article is organized as follows. First, an overview of the theories and empirical findings on the effect of work and family time claims on burnout. Second, the theories and findings regarding the effect of flexible work arrangements on burnout are introduced. Based on these theories and empirical findings, we predict the moderating effect of flexible work arrangements on the effect of work and family time claims on burnout. Figure 1 shows the corresponding theoretical model.

### Work and Family Roles and Burnout: Competing Hypotheses

#### Role Conflict Theory

In theorizing on the relationship between work and family time claims and burnout, two theories are relevant: the role conflict and the role enrichment theories (24, 27, 29). As stated above, role conflict theory and the enrichment approach arrive at different predictions. However, the two theories have in common that the individual has to fulfill different roles in the work and family spheres. The number of roles may differ between individuals and ranges from employee and coworker in the work-sphere to husband/wife, parent, and single in the family or household-sphere. It is in combining these roles from both spheres, or even within each sphere, that the two theories diverge.

Conflict theory is based on the assumption that time and energy are limited resources. For example, spending time in one sphere, the family implies less time spent in other spheres, such as work. Consequently, combining the demands pertaining to the different roles may prove conflicting. The strain caused by this conflict then causes low levels of psychological wellbeing (24, 26). Similarly, conflict theory can be used to predict burnout. When both the work and family roles pose high time claims on the individual, the combination of multiple roles becomes more complex, consequently increasing the likelihood of a person experiencing high levels of burnout. Empirical studies have shown that high work and family time claims lead to higher levels of burnout (16–18, 25, 32). Specifically, the total number of hours worked, including overtime and work pressure, is positively related to burnout and poor mental health (16, 17, 34, 35). Regarding the household, the number of children, performing household chores, and having young children are all positively related to burnout (17). The indicators of work and family time claim this study focuses on are the total of hours spent working and the total of hours spent on household chores and childcare. The impact on burnout may be particularly strong for those with traditional gender role ideology (36, 37).

#### Enrichment Theory

As a response to the conflict theory, the enrichment approach has been proposed (24, 29). The rationale behind this approach is that having multiple roles “enriches” or aids an individual in combining the multitude of roles they occupy. Specifically, as opposed to the conflict theory, it is argued that having a variety of roles is beneficial to the individual, and what is perceived as demand in conflict theory is perceived as a resource in the enrichment approach. Following (17), there are three mechanisms by which the multiple roles may benefit the individual. First, the social support an individual may receive from the family is beneficial to the individual. Second, participating in family life may in itself be rewarding and fulfilling. More specifically, this fulfillment may function as a counterbalance for strains related to long working hours or the fulfillment of multiple roles. Third, engaging in family life may lead to developing new skills and knowledge that the individual may apply and benefit from when at work and vice versa. As mentioned above, the extent to which each of the mechanisms has an effect may depend on the actual time spent in each of the spheres in which one occupies a role. For example, Van Dyne et al. (38), on the social support one may receive from colleagues, argue that less overall contact reduces social support.

Following the different arguments from role conflict theory and enrichment theory, we make the following competing hypotheses:

*H1a (role conflict theory): The higher the total hours spent working, the higher the level of burnout*.
*H1b (role conflict theory): The higher the total hours spent in the household, the higher the level of burnout*.
*H2a (enrichment theory): The higher the total hours spent working, the lower the level of burnout*.
*H2b (enrichment theory): The higher the total hours spent in the household, the lower the level of burnout*.

### Flexibility, Burnout, and Moderation

Whether and to what degree work and family time claims affect burnout may depend on contextual factors; among others, burnout has been one of the incentives for implementing flexible work arrangements on the organizational level (22). Much research has been done on the effect of flexible work arrangements on outcomes related to burnout, such as stress and time pressure. Despite the literature providing theoretical reasons to suspect a moderating role of flexibility in the work and household time claims and burnout relation, only one study explicitly draws on these arguments in studying this mechanism (30). We elaborate on these theoretical arguments in the following.

The literature is not univocal concerning exactly how flexibility may affect this link between family and work time claims and burnout. One line of argumentation states that flexible work arrangements lead to an increase in the pressure or stress resulting from work and family time claims [e.g., (12, 14, 24, 27)], whereas another line of argumentation states the opposite [e.g., (30, 32)].

First, stemming from the work-family border theory, one line of argument theorizes that flexible work arrangements increase the pressure and stress resulting from work and family demands (39). Based on the concept of role blurring, the argument put forward is that flexible work arrangements threaten a clear distinction between the work sphere and the family sphere (24, 40). For example, teleworking makes working at home possible, decreasing the physical boundaries between being at work and being at home. The result of which is a blurring of roles, causing work and family duties to converge such that individuals perform both simultaneously. The increased distractions and interruptions across tasks from the two domains caused by this multitasking are likely to increase stress and conflict (27). Although Voydanoff (27) finds that regularly doing work at home is not related to work and family conflict and perceived stress, bringing work home is related to work and family conflict and perceived stress, and receiving job contacts at home is connected to work and family conflict.

Furthermore, as shown earlier, enrichment theory argues that fulfilling roles in multiple spheres provide the individual with valuable resources, such as social support, fulfillment, and new skills and knowledge. Moreover, reaping the benefits of these resources may reduce burnout complaints (24, 29). One of these resources consists of the social support one may receive from interaction with colleagues. Focusing on the relationship between flexibility and collegiality, some researchers argue that flexible work arrangements decrease the social support employees receive from their colleagues (14). The central notion of this statement is that for an individual to develop a bond with colleagues that constitutes actual support and fulfillment, the interaction between employee and co-worker is required. Flexible work arrangements, then, are argued to decrease the opportunities for such interaction: flexible working hours reduce the chance that employers work the same hours, and telecommuting is claimed to reduce the overall time spent at work (28). This reduction in overall contact leads to less collegiality, consequently decreasing the supportive benefits associated with the role occupied in the work sphere. Research confirms the negative effect of working flexible work hours on collegiality (14). Telecommuting, however, is not found to have a negative relationship with collegiality (14).

In total, the former lines of argumentation provide theoretical grounds for the prediction that through an interaction with the effect of work and family time claims on burnout, people that use flexible working arrangements will experience higher levels of burnout. Role blurring has been related to stress and feelings of pressure (27). Since these are aspects of burnout, we may assume that a similar interaction of flexible work arrangements on the effect of work and family time claims on burnout will be found. Moreover, with regard to the theory regarding collegial social support, the reduction in social support is associated with an increase in the likelihood of burnout complaints (12). Thus, we can formulate the following hypotheses depending on our hypotheses about the effects of housework and paid work on burnout:

*H3a: If the long hours of housework and paid work increase burnout, such effects are more pronounced among employees who use flexible work arrangements*.
*H3b: If the long hours of housework and paid work decrease burnout, such effects are less pronounced among employees who use flexible work arrangements*.

Second, arguing in the opposite direction, many scholars state that flexible work arrangements interact negatively with the effect of work and family time claims on stress and pressure [Refer to (23, 41) for a meta-analytic overview]. The central idea behind this line of argument is that flexible work arrangements are regarded as resources at the workplace and provide opportunities for improving the management of work and family time claims (32, 42–44). Whether workload “causes” burnout is contingent on the degree to which combining work with responsibilities within the household, i.e., the family role, poses difficulties (24). Flexible work arrangements provide the opportunities needed to effectively manage work and family time claims, subsequently facilitating the fulfillment of multiple roles. For example, dividing tasks, like taking the kids to school, is more accessible when the work schedule can be adjusted to meet these requirements.

In summary, the former line of argumentation provides theoretical grounds for the prediction that through interaction with the effect of work and family time claims on burnout, people that use flexible working arrangements will experience lower levels of burnout. This provides us with competing expectations as to how flexibility may moderate the link between work and family time claims and burnout. In the following, these competing predictions will be empirically tested. Thus, we can formulate the following hypotheses depending on our hypotheses about the effects of housework and paid work on burnout:

*H3c: If the long hours of housework and paid work increase burnout, such effects are less pronounced among employees who use flexible work arrangements*.
*H3d: If the long hours of housework and paid work decrease burnout, such effects are more pronounced among employees who use flexible work arrangements*.

## Methods

### Data

The data are taken from the Time Competition Survey conducted in 2003 among employees at 30 Dutch firms. The purpose of the survey was to study the causes of and solutions to work-home interference (33). The type of industries covered was representative of the Dutch economy, although the service sector was somewhat overrepresented. Large organizations were also over-sampled. Home interviews were conducted with 1,114 employees and, where possible, with their partners, resulting in a response rate of 28%. Of the 3,970 employees contacted, 39% agreed to participate. Each employee was subsequently contacted at home to make an appointment for the home interview. Of the employees approached at home, 28% were not interviewed in the end, usually because the partner refused to cooperate. Analyses showed that households not willing to cooperate did not differ from those willing to join the research in terms of background characteristics (e.g., gender, education, work hours) (33). As the sampling method consisted of contacting organizations and gaining access to employees through them, employees suffering long-term burnout may be underrepresented in the sample. After excluding respondents who had a missing value on one of the variables included in the analysis, we had information on 1,058 employees.

Employees were interviewed at home and were asked to answer a written questionnaire. The interview and the written questionnaire consisted of closed questions about the respondents' family situation and work characteristics. The interviews at home lasted an average of 90 min for couples and 1 h for singles. In addition, respondents filled in a time diary during the week before the interview reporting how many hours they spent cooking, cleaning, childcare, sleeping, leisure time in groups, commuting, and working. Respondents were instructed to report how much time they had spent on each activity every evening in hours. All variables are taken from the oral interview and the time diary, except the burnout items taken from the written questionnaire, and both singles and parents are considered.

### Measures

#### Dependent Variable: Burnout

We operationalized burnout as emotional exhaustion, as this is considered the main, dominant, and most significant dimension of burnout (45). Three items measured burnout: “I feel used up at the end of the working day,” “I feel mentally exhausted because of my job,” and “I feel tired when I get up on a working day” (answer categories ranged from 1 = daily to 7 = never). A reversed scale was constructed from the mean of the three items, where higher scores on the scale indicate higher levels of burnout. The scale's reliability was high (Cronbach's α = 0.86). Further analysis shows that the distribution of the dependent variable is generally normal and is only slightly right-skewed (skewness = 0.3).

#### Work and Household Time

Household time was operationalized as hours spent on household chores and childcare per week. Respondents reported how many hours they spent buying groceries, cooking, tidying up, cleaning, keeping household accounts, doing repairs, taking care of children, and accompanying children. Work time was measured in the total number of hours worked per week. Both variables contain hours reported in the time diary, but as the time diary had missing values (*n* = 1,007), the non-time diary, estimated hours spent on the same tasks per week was used instead. We also adjusted extreme values, more than the total hours in a week, on time spent in the household for 5 cases by replacing the time diary estimate with the asked, retrospective question on time use. This was not available for one case, and here the mean time spent in the household for parents was imputed. It should be noted that although actual housework hours and work hours are highly related to family and work time claims, they may be different in subtle dimensions that are worth further investigation in future research.

#### Moderator: Flexibility

We include two indicators of flexibility: flextime and flexplace. Flextime is measured with one Likert item ranging from 1 to 5, based on “I can control when I start and get off from work,” with higher scores indicating higher flextime. We also measured flexplace using one 5-point Likert item, where respondents were asked “to what extent the employee would be able to work from home”. The limited research that addresses the perceived vs. actual use of flexible work arrangements in explaining burnout finds that the perceived measures are a stronger predictor of burnout [refer to (46):351, for a discussion)]. Although flexible time and flexible place have been widely studied separately, this study includes both variables in the model to examine the net effect of each flexible working arrangement.

#### Control Variables

##### Family Characteristics

We measured family characteristics by two indicators: the presence of a partner and a categorical variable for children. The presence of a partner was operationalized as employees who were married or living together with a partner, coded 1; others were coded 0. Having a partner has been associated with lower levels of burnout (12, 31). To account for the presence of a child, we entered a categorical variable coded 1 if there is a child younger than 6 in the household, coded 2 if the children in the household are older than 6, keeping those without a child and no children in the household as a reference category. The presence of a young child in the household has been associated with higher levels of burnout (17).

##### Demographic Characteristics

Gender was entered as a dummy variable, 0 (men) and 1 (women). Age was measured in years as a continuous variable. Respondent's education was measured on an 11-point scale ranging from 1 (not completed any education) to 11 (completed a Ph.D. degree). The direction and effect of these variables have not been univocal in the empirical studies of burnout, yet it is arguably necessary to control for these demographic features (12, 17, 30).

##### Work Characteristics

Work pressure was measured on a Likert scale using the three items “I always have a lot of work to do,” “I must work very fast,” and “I work under time pressure” (Cronbach's a = 0.743, answer categories ranging from 0 = never to 5 = daily). This variable is one of the strongest predictors of high burnout (12, 17). The supervisory position was entered as a dummy, 0 if the employee was in a staff position and 1 if in a management or supervisory position. This variable was entered to account for the different qualitative work demands pertaining to these job positions. Further analyses show that Variance Inflation Factor is below two for all variables, suggesting no multicollinearity in the model.

### Data Analysis

For the analysis, we ran four OLS regression models. First, we entered a baseline model with controls only to see whether the main variables have an effect when controlled for family, work, and demographic characteristics. Second, to analyze hypotheses 1a, 1b, 2a, and 2b, work and household time were added separately to the base model. Third, we added the two flexibility variables to assess any main effect of flextime and flexplace. Finally, the interaction terms between the two time variables and the two flexibility variables were added in the fourth model to test whether and how flexibility moderates the relationship between work and household time claims and burnout. To evaluate the fit of the different models, we provide both the total R2 and the Aikake Information Criterion (AIC), where a low AIC indicates a relatively better fit (47). As the employees are clustered in organizations, we employed a cluster correction to the regression models. The cluster correction controls for the fact that employees of one organization may be more similar to one another than employees of different organizations by adjusting the standard errors. To test the interaction effects of flexibility on the relationship between work and family time and burnout, we calculated the cross-products of work and family time with flextime and flexplace, respectively. The four variables were all centered at their means, resulting in four interaction terms. The descriptive statistics for all the variables entered in the analysis are shown in Table 1.

**Table 1:** Descriptive statistics for variables used (*N* = 1,058).

**Variables**	**Mean^a^**	**SD**	**Range**
Burnout	3.33	1.43	[1, 7]
Work time^b^	35.17	11.67	[0, 68]
Household time^b^	23.52	14.59	[0, 85]
Flextime	3.87	1.42	[1, 5]
Flexplace	2.85	1.36	[1, 5]
Work Pressure	3.09	0.85	[1, 5]
Children			[0, 2]
(Ref: no child)	-	-	
Children <6 years	22.8^a^	-	
Older children	27.83^a^	-	
Partner Present	74.5^a^	-	[0, 1]
Age	40.14	9.01	[17, 62]
Education	6.99	2.19	[0, 10]
Supervisory position	40.5^a^	-	[0, 1]
Gender (Female=1)	46.7^a^	-	[0, 1]

a *The percentage is provided for dummy and categorical variables*;

b *In total hours per week*.

## Results

Table 2 presents the results of the four models of the determinants of burnout, with standard errors adjusted for clustering. In Model 1, in which only control variables are included, being in a supervisory position and being older are both associated with lower levels of burnout. High work pressure is significantly related to higher burnout. We do not find any significant effect of having a young or old child relative to having no children, education, presence of a partner, and gender.

**Table 2:** Unstandardized coefficient estimates for OLS regression predicting burnout (*N* = 1,058).

**Variables**	**M1**	**M2**	**M3**	**M4**
Age	−0.013^*^	−0.014^*^	−0.014^*^	−0.013^*^
	(0.056)	(0.005)	(0.005)	(0.005)
Gender (Female=1)	−0.018	−0.018	−0.017	−0.012
	(0.104)	(0.099)	(0.097)	(0.095)
Education	0.024	0.024	0.029	0.027
	(0.026)	(0.028)	(0.029)	(0.028)
Supervisory Position	−0.181^*^	−0.186^*^	−0.189^*^	−0.193^*^
	(0.083)	(0.087)	(0.087)	(0.086)
Partner Present	−0.123	−0.114	−0.116	−0.130
	(0.129)	(0.138)	(0.137)	(0.140)
Children (Ref = No child)	-	-	-	-
Young child (<6)	−0.111	−0.163	−0.160	−0.105
	(0.129)	(0.148)	(0.149)	(0.142)
Older children (≥6)	−0.156	−0.177	−0.170	−0.161
	(0.127)	(0.128)	(0.130)	(0.135)
Work Pressure	0.542^***^	0.538^***^	0.537^***^	0.536^***^
	(0.049)	(0.048)	(0.049)	(0.049)
Work Time		0.003	0.003	0.002
(Hours per week)		(0.005)	(0.005)	(0.005)
Household Time		0.003	0.004	0.002
(Hours per week)		(0.004)	(0.004)	(0.003)
Flextime			0.024	0.032
			(0.028)	(0.029)
Flexplace			−0.037	−0.035
			(0.032)	(0.034)
Household Time ^*^ Flextime				−0.006^*^
				(0.002)
Work Time ^*^ Flextime				−0.005^*^
				(0.002)
Constant	2.267^***^	2.302^***^	2.281^***^	2.234^***^
	(0.374)	(0.374)	(0.391)	(0.383)
AIC	3633.541	3636.074	3638.793	3632.994
Total R^2^	0.128	0.129	0.130	0.142
Total F Statistics	19.09^***^	20.86^***^	17.63^***^	29.78^***^

*1) Values for flextime, flexplace, household and work time are centered at their means; 2) Figures in parentheses are standard errors adjusted for clustering on firms; 3)*

* *p < 0.05*,

^**^p < 0.01,

*** *p < 0.001, in a 2-sided test*.

In Model 2, the number of hours involved in family and work is included. Although the total R^2^ increases, the AIC increases as well (Δ: 2.53), suggesting that Model 2 does not have a better fit than the controls-only model. Moreover, the main effects of hours worked and hours spent on various tasks in the household are not significant in explaining burnout. Contrary to the expectation that time spent at work or in the family would predict burnout, either positively (H1a and H1b) or negatively (H2a and H2b), our analysis finds no significant relationship between time and burnout.

Contrary to the expectation that time spent at work or in the family would predict burnout, either positively (H1a and H1b) or negatively (H2a and H2b), our analysis finds no significant relationship between time and burnout. In Model 2, the number of hours involved in family and work is included. Although the total R2 increases, the AIC increases as well (Δ: 2.53), suggesting that Model 2 does not have a better fit than the controls-only model. Moreover, the main effects of hours worked and hours spent on various tasks in the household are not significant in explaining burnout.

Further analyses find that no significant relationship could be found even when only the time variables were used to predict burnout. Only when we did not adjust for clustering on firms could a significant relationship between work time and burnout be found (*p* < 0.05, 2-sided). However, no significant association between time in the household and burnout could be found. This suggests that work time is dependent on the firm, which is further supported by a Pearson's Chi^2^ test of the cross-tabulation between firms and work time: Chi^2^(145) = 284.45, *p* < 0.001, 2-sided).

In Model 3, the two flexibility variables were added. The results indicate that the model fit did not improve, as the AIC was the highest among all models. Moreover, the two variables are also not significant in explaining levels of burnout. Turning to Model 4, where the interaction effects are implemented, we see that the AIC is the lowest among all four models, indicating a better fit than all the other models. However, it should be noted that as the difference between the lowest (M4) and second-lowest (M2) AIC is not larger than 4, the difference in fit is not substantial. Only the significant interactions are provided in the table; we see that only the flextime interactions were significantly related to the number of hours spent at work and in the household (*p* < 0.05 for both). This supports hypothesis 3c that the positive impacts of housework and paid work hours on burnout are less pronounced among employees who use flexible time arrangements. To illustrate and interpret these interaction effects, we provide two figures described in the following.

**Figure 2:**
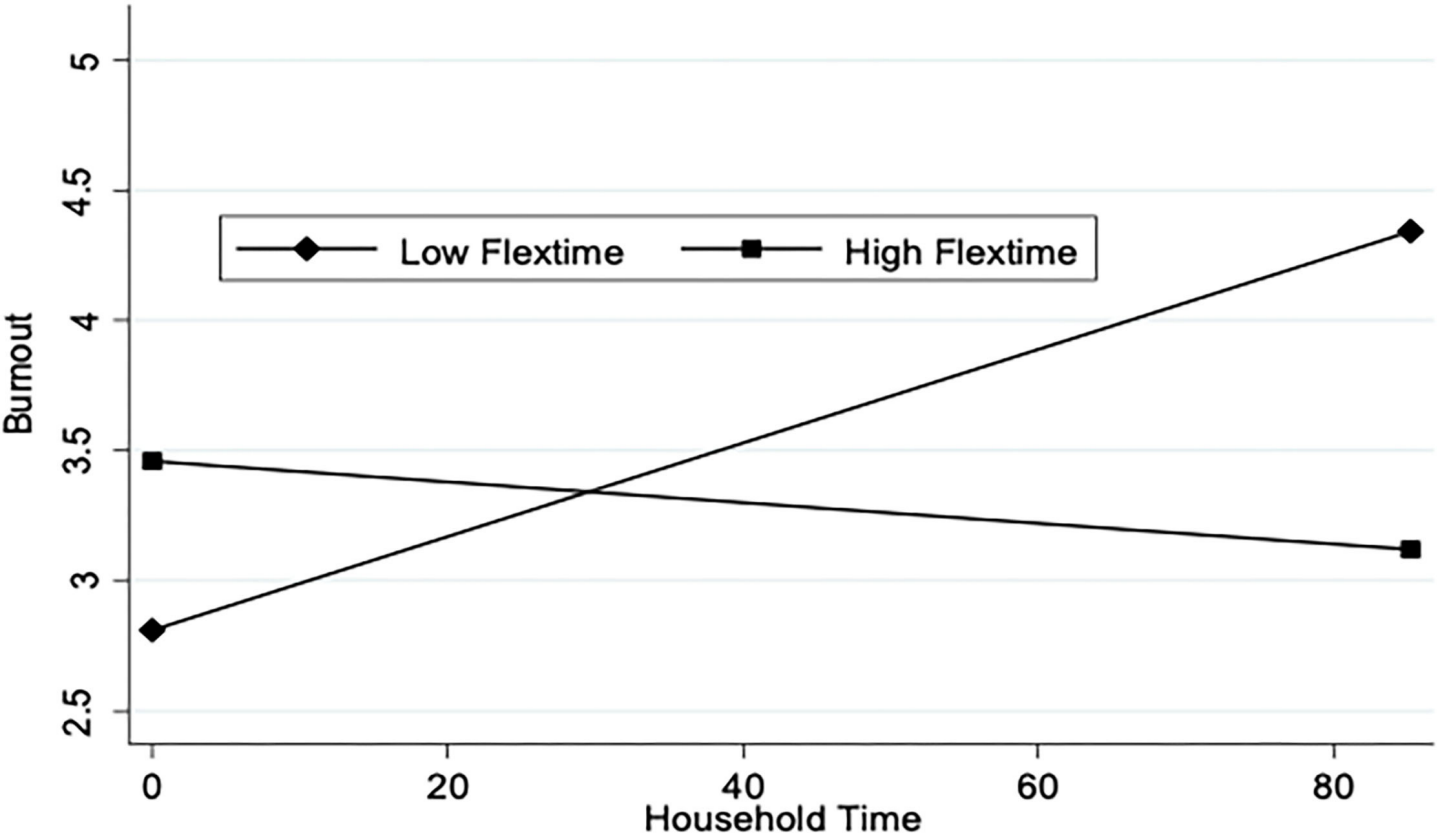
Household time and predicted burnout by flextime.

Figure 2 indicates that when time spent in the household increases, low flextime is associated with a higher increase in burnout. However, when flextime is high, household time is associated with about the same level of burnout despite an increase in time spent in the household. This finding supports the notion that high flextime is associated with lower levels of burnout even when there is an increase in time spent in the household.

**Figure 3:**
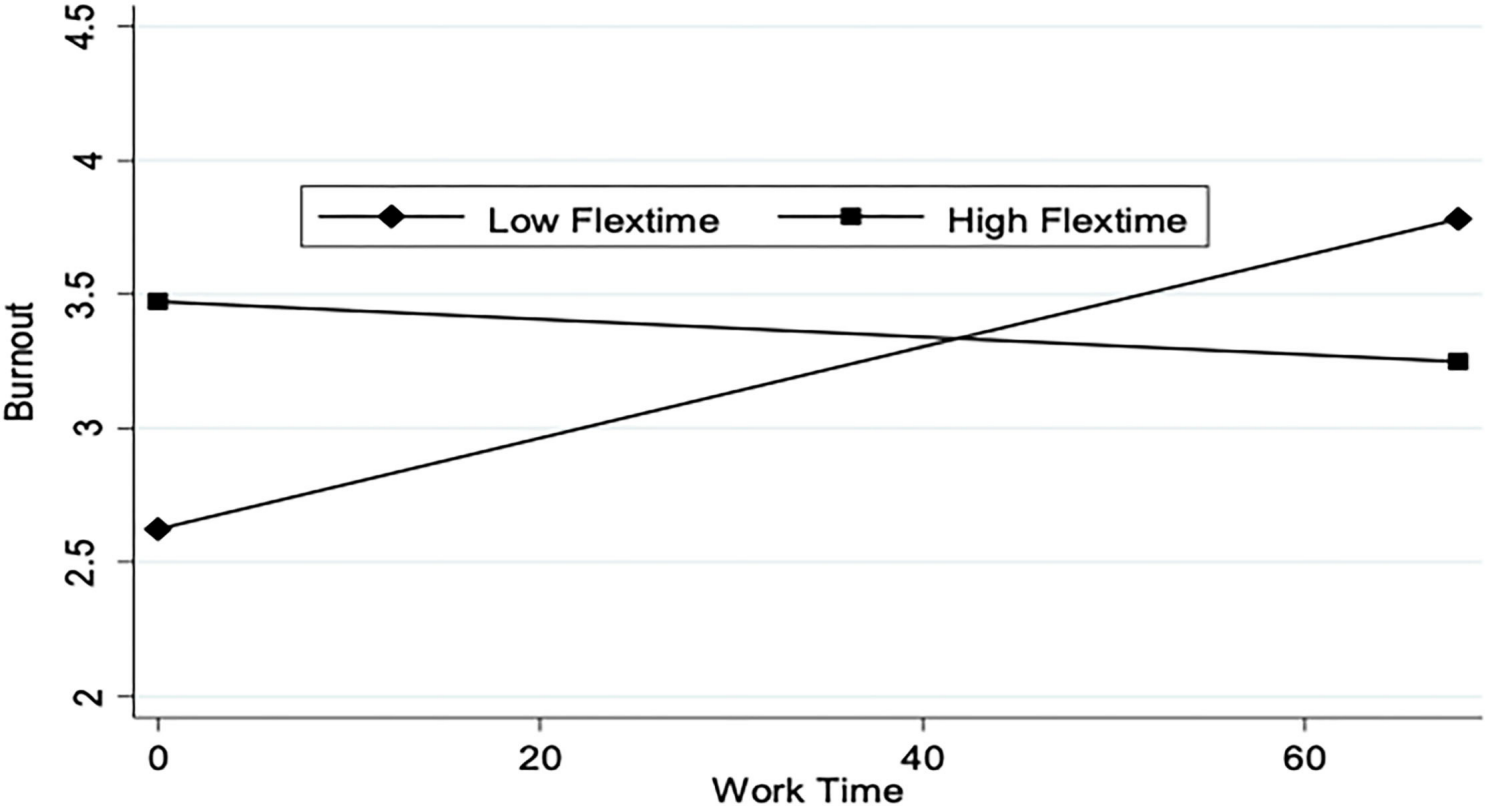
Work time and predicted burnout by flextime.

Figure 3 shows a similar relationship for the interaction between time at work and flextime. As the number of working hours increases, burnout increases for those with low flextime. Whereas the level of burnout remains about the same for those with high flextime, irrespective of the increase in working hours. Thus, our results suggest that high perceived flextime moderates the impact of high work time on burnout.

To ensure the robustness of the results, we have used the median burnout score as the cutpoint and recoded burnout into a binary variable with 0 indicating low burnout (lower than the median score) and 1 indicating high burnout. Additionally, then we repeated our main analyses using logistic regression models in Supplementary Table A1 in Appendix. Overall, the results are consistent with our main findings that the higher an individual's work and family time claims, the more this person benefits from having flexible working hours. This suggests that our results are robust to alternative variable specifications.

## Discussion

This study aimed to study how flexible work arrangements interact with work and family time claims to affect burnout. We conclude that flexible work arrangements and work and family time claims fail to account for burnout. However, the combination of work and family time claims and flexible work arrangements does provide a substantive prediction of burnout.

First, we looked at the relationship between burnout and work and family time claims. In theorizing this relationship, we focused on arguments derived from conflict theory and the enrichment approach. Contrary to (17), we found no main effects of time claims on burnout, and our findings do not univocally support either one of these theories. However, some scholars have argued that these conflict and enrichment processes may coincide (17, 48, 49). As more time is spent on fulfilling family and work roles, according to conflict theory, friction increases, leading to higher levels of burnout. The enrichment approach then argues in the opposite direction. As more time is spent on fulfilling work and family roles, the gains an individual receives from occupying these roles increase, consequently decreasing burnout. However, if these processes co-occur, these effects might cancel each other out, which may account for the non-significant main effects. Thus, although our findings do not support either of the two theories, the present study does not lead us to refute the theories either.

Second, we looked at the effect of flexible work arrangements on burnout. More specifically, we looked into how flexible work arrangements affect the relationship between work and family time claims and burnout. Burnout in the current study is not directly related to flexible working arrangements. However, the results support the predictions stemming from the time management argument that flexible work arrangements allow people to cope better with fulfilling multiple roles (23, 41). As the total hours spent in the work and family sphere increase, the more difficult a satisfactory fulfillment of these multiple roles becomes. Managing working hours, i.e., flextime alleviates these difficulties, consequently decreasing burnout. This only seems to hold for flextime, as we did not find that flexplace significantly moderated the association between work and household time claims and burnout. A possible explanation for this is that within the group of flexplace-users, we can differentiate between different sub-groups for which the effect of flexplace on burnout may differ. For example, one may argue that for those with longer commuting time, the need and impact of flexplace are more potent than for those with short commuting time. However, the inclusion of these characteristics needed to differentiate between sub-groups of flexplace-users is beyond the scope of this research. Also, previous research shows that employees who request flexible place arrangements are more likely to suffer discrimination from employers than those who request flexible time arrangements (50). The higher flexibility stigma of flexible place arrangements may help explain why they do not have moderating effects.

### Limitations, Future Research, and Implications

Some limitations of the present study should be pointed out. First, as the research design is cross-sectional, no causal relationship conclusions can be drawn. Second, only the emotional exhaustion dimension of burnout is measured. Although this dimension is shown to be the main feature of burnout (45), burnout's “depersonalization” and “diminished personal accomplishment” dimensions are not addressed here. Despite these limitations, the study's strengths are that we have detailed information on both work and family variables at the employee level and the usage of a time diary to get detailed and reliable information on the actual time use of the employee (51).

One direction for future research would be to establish the exact relationship between family and work time variables and measures of flexibility and their consequent effect on burnout in a longitudinal research design. For example, one may study how high time claims from work and family affect the use of flexible work arrangements and how this may alleviate burnout. This would lead to inferences on whether and how employees manage their time between work and family and how they may employ flexible work arrangements to accommodate fulfilling multiple roles. Another avenue for future research could be to study other family-supportive policies (52), besides flextime and flexplace, and how these other policies, such as civic participation, may moderate the relationship between time claims and burnout (53). In addition, although this research was conducted in the Netherlands, a developed country, the results in this study also hold significant implications for developing countries in terms of developing employee-friendly workplace policies. Future research should also pay more attention to developing countries where employees usually work long hours and do not have access to flexible working arrangements (5).

The findings of this study can be helpful in improving the effective deployment of flexible work arrangements. To maximize the gains from implementing, for example, family-friendly policies, it is crucial to know who will benefit most from a particular arrangement. Organizations that have high demands regarding time claims may want to provide more flexible work schedules to help their employees cope with combining the work time claims with those of other spheres such as the family.

In conclusion, this study contributes to the previous literature by studying the interaction of flexible work arrangements with work and family time claims on burnout. Individuals with high family and work time claims appear to benefit more from managing their working hours than those with lower time claims. It seems that when flexible work arrangements influence burnout, it is in combination with time claims and vice versa. In general, the results support the proposition that the relationship between work and family time claims and burnout differs for individuals with different levels of flexible work arrangements. The findings of this study can be helpful in improving the effective deployment of flexible work arrangements. To maximize the gains from implementing, e.g., family-friendly policies, it is crucial to know who will benefit most from a particular arrangement. Organizations that have high demands regarding time claims may want to provide more flexible work schedules to help their employees cope with combining the work time claims with those of other spheres such as the family.

## Data Availability Statement

The original contributions presented in the study are included in the article/supplementary material, further inquiries can be directed to the corresponding author/s.

## Ethics Statement

The studies involving human participants were reviewed and approved by Ethics Review Board of the Faculty of Social & Behavioral Sciences, Universiteit Utrecht. The patients/participants provided their written informed consent to participate in this study.

## Author Contributions

SL designed the research and revised the paper. SL, JtB, and MHK analyzed and interpreted the data. All authors wrote the manuscript, contributed to the article, and approved the final manuscript.

## Funding

The research was financially supported by a grant from the National Social Science Fund of China (Grant No. 20CSH019) awarded to SL.

## Conflict of Interest

The authors declare that the research was conducted in the absence of any commercial or financial relationships that could be construed as a potential conflict of interest.

## Publisher's Note

All claims expressed in this article are solely those of the authors and do not necessarily represent those of their affiliated organizations, or those of the publisher, the editors and the reviewers. Any product that may be evaluated in this article, or claim that may be made by its manufacturer, is not guaranteed or endorsed by the publisher.
