# Supplementary material for: Burnout and Its Antecedents: Considering Both Work and Household Time Claims, and Flexibility in Relation to Burnout
Source: Front Public Health. 2022 May 10;10:863348. doi: 10.3389/fpubh.2022.863348 (PMC9128481; doi:10.3389/fpubh.2022.863348)
Supplement: Supplementary file 1 [file Data_Sheet_1.docx]

Appendix

**Table A1: Unstandardized Coefficient Estimates for Logistic Regression Predicting Burnout (N=1,058)**

| Variables | M1 | M2 | M3 | M4 |
| --- | --- | --- | --- | --- |
| Age | -0.019** | -0.019** | -0.019** | -0.018* |
|  | (0.006) | (0.006) | (0.006) | (0.007) |
|  |  |  |  |  |
| Gender (Female=1) | 0.049 | 0.086 | 0.087 | 0.102 |
|  | (0.13) | (0.13) | (0.135) | (0.135) |
|  |  |  |  |  |
| Education | 0.021 | 0.017 | 0.027 | 0.026 |
|  | (0.033) | (0.035) | (0.036) | (0.036) |
|  |  |  |  |  |
| Supervisory Position | -0.156 | -0.169 | -0.172 | -0.174 |
|  | (0.137) | (0.144) | (0.142) | (0.143) |
|  |  |  |  |  |
| Partner Present | -0.203 | -0.202 | -0.206 | -0.220 |
|  | (0.194) | (0.191) | (0.192) | (0.197) |
|  |  |  |  |  |
| Children (Ref = No child) | - | - | - | - |
| Young child (<6) | -0.024 | -0.119 | -0.113 | -0.063 |
|  | (0.226) | (0.247) | (0.250) | (0.241) |
|  |  |  |  |  |
| Older children (>=6) | -0.116 | -0.155 | -0.142 | -0.142 |
|  | (0.181) | (0.173) | (0.173) | (0.184) |
|  |  |  |  |  |
| Work Pressure | 0.588*** | 0.575*** | 0.575*** | 0.579*** |
|  | (0.064) | (0.066) | (0.068) | (0.067) |
|  |  |  |  |  |
| Work Time |  | 0.011 | 0.011 | 0.010 |
| (Hours per week) |  | (0.007) | (0.007) | (0.007) |
|  |  |  |  |  |
| Household Time |  | 0.007 | 0.007 | 0.006 |
| (Hours per week) |  | (0.005) | (0.005) | (0.005) |
|  |  |  |  |  |
| Flextime |  |  | 0.042 | 0.051 |
|  |  |  | (0.033) | (0.037) |
|  |  |  |  |  |
| Flexplace |  |  | -0.072 | -0.066 |
|  |  |  | (0.056) | (0.057) |
|  |  |  |  |  |
| Household Time * Flextime |  |  |  | -0.006+ |
|  |  |  |  | (0.003) |
|  |  |  |  |  |
| Work Time * Flextime |  |  |  | -0.006+ |
|  |  |  |  | (0.003) |
|  |  |  |  |  |
| Constant | -0.863 | -0.809 | -0.864 | -0.944* |
|  | (0.444) | (0.448) | (0.449) | (0.437) |
| AIC | 1469.746 | 1466.711 | 1468.739 | 1468.739 |
| Total R^2^ | .054 | .054 | .057 | .063 |

*Note: 1) Values for flextime, flexplace, household and work time are centered at their means; 2) Figures in parentheses are standard errors adjusted for clustering on firms; 3) + p < .1,* p < .05, ** p < .01, *** p < .001, in a 2-sided test.*
